# Entry Points, Barriers, and Drivers of Transformation Toward Sustainable Organic Food Systems in Five Case Territories in Europe and North Africa

**DOI:** 10.3390/nu17030445

**Published:** 2025-01-25

**Authors:** Rita Góralska-Walczak, Lilliana Stefanovic, Klaudia Kopczyńska, Renata Kazimierczak, Susanne Gjedsted Bügel, Carola Strassner, Benedetta Peronti, Amina Lafram, Hamid El Bilali, Dominika Średnicka-Tober

**Affiliations:** 1 Department of Functional and Organic Food, Institute of Human Nutrition Sciences, Warsaw University of Life Sciences, Nowoursynowska 159c, 02-776 Warsaw, Poland; klaudia_kopczynska@sggw.edu.pl (K.K.); renata_kazimierczak@sggw.edu.pl (R.K.); dominika_srednicka-tober@sggw.edu.pl (D.Ś.-T.); 2 Section of Organic Food Quality, Faculty Organic Agricultural Sciences, University of Kassel, Nordbahnhofstrasse 1a, 34125 Witzenhausen, Germany; l.stefa@uni-kassel.de; 3 Department of Nutrition, Exercise, and Sports, University of Copenhagen, Rolighedsvej 26, 1172 Frederiksberg, Denmark; shb@nexs.ku.dk; 4 Department of Food-Nutrition-Facilities, FH Münster University of Applied Sciences, Corrensstraße 25, 48149 Muenster, Germany; strassner@fh-muenster.de; 5 Council for Agricultural Research and Economics—Research Centre for Food and Nutrition (CREA—Food and Nutrition), Via Ardeatina, 12010 Rome, Italy; benedetta.peronti@crea.gov.it; 6 Department of Life Sciences, Faculty of Sciences, Ibn Tofail University, Kenitra 14000, Morocco; amina.lafram@uit.ac.ma; 7 International Centre for Advanced Mediterranean Agronomic Studies (CIHEAM-Bari), Via Ceglie 9, 70010 Valenzano, Italy; elbilali@iamb.it

**Keywords:** sustainability, mixed methods, organic diet

## Abstract

**Background:** The organic sector is often suggested as a lever with a potential for contributing to the three dimensions of sustainability: social, environmental, and economic. This study aims to investigate selected organic initiatives and organic food sectors in different locations, such as capital cities, rural areas, and the bio-district in SysOrg project consortium, in the Warsaw municipality in Poland, North Hessia region in Germany, Cilento bio-district in Italy, Kenitra province in Morocco, and Copenhagen municipality in Denmark to uncover the diverse drivers, barriers, and entry points to enable a transformation process to resilient and sustainable organic food systems. **Methods:** Following the methodology of the SysOrg project, this study relied on the following mixed data collection methods: quantitative (a household survey distributed among citizens) and qualitative (semi-structured interviews with organized initiatives). **Results:** The results demonstrate that, despite being in different stages of development in the investigated territories, the organic sector is challenged by similar barriers (e.g., undeveloped market, regulatory/budgetary constraints, and lack of knowledge and awareness) and benefits from analogous drivers (e.g., awareness and education, community support, and incentives). **Conclusions:** Those similarities, but also analyses of their differences and origins, allowed us to establish critical entry points for the development of a sustainable organic food system, e.g., promoting organics through a top-down approach, providing training and education, reducing information delay, popularizing negative feedback, strengthening the effectiveness of a given incentives scheme by tailored nudging mechanisms, establishing country/regional specific traditional frames, making the system more inclusive, building organic communities, and awareness-building.

## 1. Introduction

The food system comprises “an interconnected web of activities, resources, and people, which involve all domains of the food value chain and more” [1]. Scientific research shows that the current food system threatens healthy and sustainable diets [2,3], population wellbeing, the environment, and the global economy [2]. Over the past decade, a sustainable food system has increasingly been acknowledged as part of the solution to the problems of natural resource degradation and human health impairment, with arguments being put forward regarding its potential to help align humanity’s activities with the United Nations’ Agenda for Sustainable Development [4,5,6].

However, to reverse the current negative trends related to the production and consumption of food and redirect the food system towards a sustainable trajectory within the planetary boundaries, a food systems transformation is required—a change that would lead to a resilient and just food system “delivering food security and nutrition for all in such a way that the economic, social and environmental bases to generate food security and nutrition for future generations are not compromised” [7], and thus would transform a food system from a primary threat to a primary solution [6].

When investigating the possible transformation avenues toward sustainable food systems, the organic path is often found in the spotlight. The organic food system is a values-driven sub-system of a broader global food system aimed at “feeding people organically” based on the four principles of organic farming—principles of health, ecology, fairness, and care [1,8,9]. According to the FAO and WHO [10], organic agriculture is a holistic production management system that has the potential to promote and enhance agro-ecosystem health, including biodiversity, biological cycles, and soil biological activity, using, where possible, agronomic, biological, and mechanical methods, as opposed to using synthetic materials, to fulfill any specific function within the system. According to the definition of IFOAM [11], organic agriculture should enhance the health of soils, ecosystems, and people. By combining tradition, innovation, and science, it should protect the environment and promote fair relationships and good quality of life for all involved. The EU Council Regulation defines organic production as “(…) an overall system of farm management and food production that combines best environmental practices, a high level of biodiversity, the preservation of natural resources, the application of high animal welfare standards and a production method in line with the preference of certain consumers for products produced using natural substances and processes” [12]. Organic farming has a history of more than 100 years and a clear vision with underlying principles that were translated into metrics [1,13]. Having originated in Europe, organic practices were adopted worldwide, with international standards and a corresponding legislative basis in place in many parts of the world, including nationwide and private regulations [13]. The potential of organic systems to result in positive outcomes across the sustainability dimensions was previously demonstrated in a number of studies [14,15,16]. Likewise, within the environmental pillar, organic practices at the operator level were suggested to perform better in the realms of resource depletion, climate change, biodiversity and landscape, water pollution, air quality, soil fertility, economic resilience, and the long-term stability of production [15,17]. Potential contributions to climate change mitigation, thanks to organic soil management practices, were also reported [18,19]. A recent systematic literature review by Hashemi et al. (2024) [20], incorporating 100 studies, showed overall lower negative impacts of organic products per unit area compared to conventional ones. Within the social and economic dimensions, a number of studies reported positive outcomes of organic systems, linked to increased employment, farmers’ income diversification, socio-economic resilience, safer working conditions on organic farms, labor rights, equity, farmers’ autonomy, improved food, nutrition security for farmers and their families, animal welfare, revitalization of rural communities, and contributions to farmers’ wellbeing [16,21,22]. Even though there is an ongoing scientific debate on whether organic agriculture should reconsider certain principles to more effectively serve as a driving force for increased food system sustainability [23], the organic food system, in its current shape, is discussed in the context of its potential to address many of the Sustainable Development Goals (SDGs), with a possible contribution to eight goals shown at the goal level [18] and most of the goals at the target level [10,24].

Since the organic sector shows potential to contribute to the three dimensions of SFSs, i.e., social (broad-based benefits for society), environmental (minimizing the negative impact of food production on the natural environment), and economic (profitable throughout) [2,3,13,25], an investigation of the organic food sector in different locations is crucial to provide insights into the diverse drivers, barriers, stakeholders, initiatives, and entry points to enable a transformation process to resilient, sustainable, and organic food systems. Thus, organic food and farming are some of the key areas investigated by the SysOrg project “Organic agro-food systems as models for sustainable food systems in Europe and Northern Africa”, which aimed to characterize and analyze five territorial cases (viz. the Warsaw municipality in Poland (WA), North Hessia region in Germany (KA), Cilento bio-district in Italy (CI), Kenitra province in Morocco (KE), and Copenhagen municipality in Denmark (CO)) regarding the four following perspectives: system transition, sustainable and healthy diet, organic food and farming, as well as food waste reduction. Doing this will help to identify how pathways to increase sustainable and organic consumption and food production could be designed.

This article focuses on the organic perspective as a hypothetical primary force for sustainable transition, using the five case territories that represent the project consortium, with each of them being in various stages of organic transformation. The research design employed in this project relied on a mixed methods approach involving both quantitative and qualitative methods: desk research, household survey (HHS), and semi-structured interviews (SSIs). This study aims to assess and compare consumers’ attitudes toward organic food and to analyze organic transition initiatives in order to derive the main drivers and barriers toward sustainable organic food systems in the project’s five case territories.

## 2. Materials and Methods

### 2.1. Conceptual Framework and Approach

**Figure 1:**
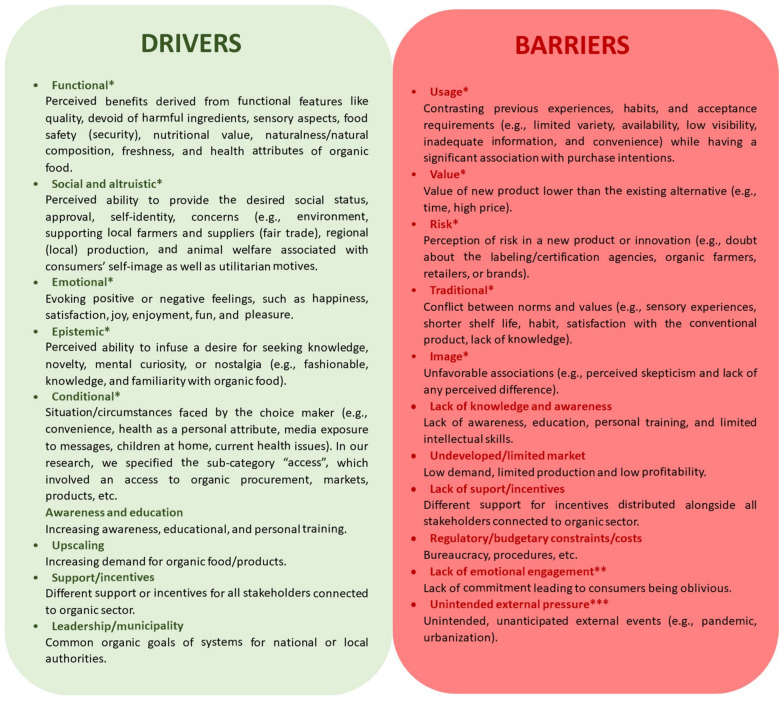
Conceptual framework of drivers and barriers selected for this paper (own elaboration) based on the reviews by * Kushwah et al., 2019 [26], ** van Geffen et al., 2020 [30], and *** Béné et al., 2020 [31].

To characterize the motives (drivers) for the organic trajectory and the root causes of the barriers in its way, we have adopted the classification from the review of Kushwah et al., 2019 [26]. The drivers are divided into the following five dimensions: functional, social, emotional, conditional, and epistemic, while the barriers, according to innovation resistance theory [27], are divided into usage, value, risk, image, and tradition barriers. Additionally, when researching dimensions other than consumption in the literature, e.g., production and distribution, we derived both drivers and barriers inductively (Figure 1).

**Figure 2:**
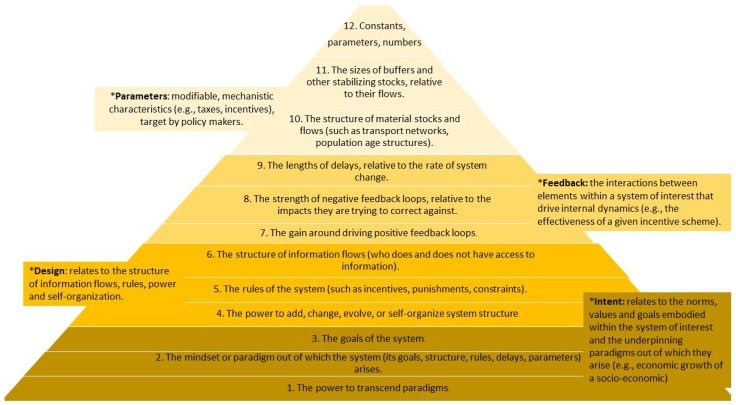
Conceptual framework of types of interventions (entry points) and system characteristics (marked with *). Pyramid structured from deeper realms of entry points to shallower, own elaboration based on Abson et al. 2017 [29]; Meadows 1999 [28].

Based on the analyses of drivers and barriers, we have established the types of interventions (entry points) that may be crucial to system change. To establish the entry points for organic FS transformation, we used Donella Meadows’ concept of leverage points [28] in complex systems, where a small shift may lead to fundamental changes in the system as a whole (Figure 2). Furthermore, to understand “the state of the system” (status quo) [28], in this case, organic FSs in five case territories, we have researched and compared the organic sector across these. However, to investigate the possible organic transition of those systems, we undertook a more epistemological approach to the notion, which was to view systems thinking as a lens through which sustainability issues, as drivers and barriers of organic transition, can be addressed [29]. Meadows’ leverage points—types of interventions (entry points)—may be structured in a pyramid, with subsequent pyramid levels reflecting the increasing effectiveness of the entry points for systemic change and, at the same time, an increasing difficulty regarding their implementation. The pyramid levels are established within four system characteristics: intent, design, feedback, and parameters. The entry points may influence a system change from deeper system characteristics (bottom of the pyramid—intent) up to shallower realms (top—parameters), but also vice versa [29] (Figure 2).

The data in this study were collected from the five case territories (WA, CO, KA, CI, and KE) of the SysOrg project and analyzed using quantitative and qualitative methods, which complemented each other and allowed for a more comprehensive analysis [32]. The quantitative part of this study was based on HHS, while the qualitative one focused on SSIs with selected organic transition initiatives. This mixed methods approach was selected to scrutinize the diverse perspectives of consumers and initiatives and to analyze and compare the organic sector in each of the case territories to gain a deeper understanding of the drivers and barriers toward a more sustainable organic food system. The quantitative and qualitative parts have been connected, and the results have been integrated. Additionally, to support these two sources (HHS and SSIs), desk research data were collected.

### 2.2. Five Case Territories at a Glance

**Figure 3:**
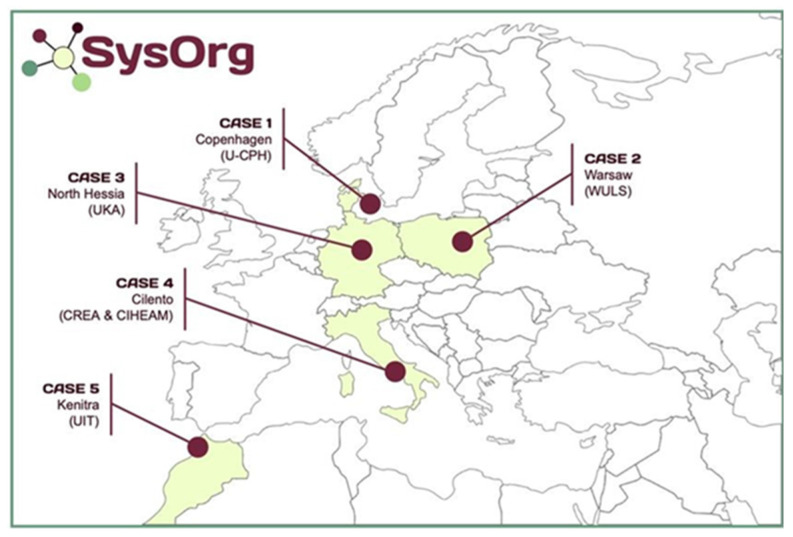
Map of the five case territories within the SysOrg project.

The research was carried out within the framework of the SysOrg project, and this publication covers all five SysOrg territorial cases with different characteristics regarding their organic sector, including status quo and dynamics (Figure 3). Territories are defined in the project guidelines based on geographic and administrative boundaries. A description of the territories is provided in Appendix A, Table A1.

### 2.3. Quantitative Approach—Household Survey

The survey questionnaire was designed by the SysOrg consortium to collect information regarding three project perspectives: diet, organic, and waste. The questions addressed, among others, sociodemographic information, food consumption frequency, the percentage of organic food consumption in the HH, the choices of organic products, and sustainable diet, and were partially based on the previously validated questionnaires, i.e., the UK Million Women Study and the European Consumers Survey on attitudes towards sustainable food (BEUC), One Bite At A Time for European consumers on attitudes towards sustainable food (2020) [33] for the diet section, while the Households Food Waste Questionnaire (HFWQ) [34] and REFRESH—Food Waste questionnaire [35] were used for the food waste section.

For this publication, questions from the survey were chosen with the aim to characterize and compare organic consumption and attitudes towards organics, and to explore the possible drivers and barriers of transition towards a sustainable organic food system in the five case territories. For that, we have analyzed the claimed percentage of organic food out of all foods consumed, the types of organic products consumed, willingness to change to more sustainable eating habits, the descriptors that the respondents associated with organic food and sustainable food, an increase in HH purchases of organic food over the past five years, and, in case of the absence of an increase, the barriers for buying organic food. The selected questions from the survey are reported in Supplementary Materials, Table S1.

The survey, initially designed in English, was translated into the languages of the studied territories and distributed in 2022 using river sampling methodology [36] through diverse social media channels (e.g., Facebook, Instagram), online sites, the CATI system (computer-assisted telephone interviews), CAWI system (computer-assisted web interviews), and face-to-face interviews. The sampling procedure differed slightly, depending on the territory’s particularities [37].

Statistical analyses were performed using Statistica software, version 13.3 PL (StatSoft Inc., Tulsa, OK, USA; StatSoft, Krakow, Warsaw). Charts were made using Microsoft Excel 2016, while the dot plots were made using R Statistical Software version number 4.1.1. The qualitative variables were presented as numbers and percentages, while the quantitative variables were presented as means with standard deviations or as medians with minimum and maximum values. Pearson’s chi-square test was used to assess the relationship between qualitative variables. Differences between independent multiple groups for the variables on the ordinal scale were assessed using the Kruskal–Wallis rank-sum test. For all tests, a *p*-value < 0.05 was considered significant.

### 2.4. Qualitative Approach—Semi-Structured Interviews

To amplify and enrich the results, semi-structured interviews with representatives of organic initiatives were conducted. In this study, we define initiatives as the entities involved within the agri-food system, applying regular actions towards sustainable development, with at least two active members and two years of operation, and located or active within the pre-defined case territory. The initiatives were selected based on their size, activities supporting organic transition, and their impact on the organic sector. For data collection, we used a three-step process as follows: (a) collecting: brainstorming and asking local actors; (b) screening: desk research and/or short interviews were conducted to gather information about each initiative, including the locality, starting date, main actors, size, novelty, and impact on the food system; and (c) mapping: semi-structured interviews with initiative informants were carried out focusing on drivers and supportive circumstances for the development of the organic sector in the respective territory, as well as challenges or barriers to their activities. Data collectors from all territories have conducted semi-structured interviews with the informants representing organic initiatives: two in CO and CI, three in KA, and four in WA and KE (see Appendix C, Table A3). The interviews were performed online via Zoom, in the participant’s original language, recorded, transcribed verbatim, and then translated into English for further analysis. The organic-related SSI questions are reported in Appendix B.

For the coding process, analysis, and graphs, the software program MAXQDA 2024 (VERBI Software, Consult Sozialforschung GmbH, Berlin, Germany) was used. To analyze the transcripts, Kuckartz’s thematic qualitative text analysis [38] was used based on the content reductive analysis [39]. The first step included the initial work with the text, marking important passages, and creating memos. The second step was developing the main topical categories, performing the first coding using the main categories, and determining the sub-categories, including the number of times that codes were mentioned. A deductive–inductive coding scheme was applied, focusing on the research questions about the main drivers and barriers towards sustainable organic food systems in the five case territories. Lastly, second coding was performed with a second coder using an elaborated categories system [40].

## 3. Results and Discussion

The characteristics of the study sample are reported in Appendix C, Table A3 (Organic Initiatives), and Appendix C, Table A4 (Household Survey respondents).

### 3.1. Organic Consumption Status Quo and Dynamics in the Territories

**Figure 4:**
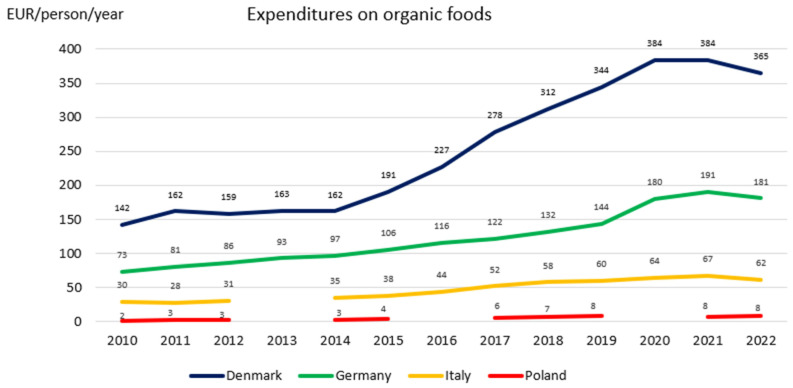
Expenditures on organic food in Poland, Denmark, Germany, Italy, and Morocco in 2010–2022. Own elaboration based on The World of Organic Agriculture, Statistics and Emerging Trends (2010–2024) from the Research Institute of Organic Agriculture FiBL, Frick, and IFOAM—Organics International, Bonn. The lack of values and corresponding lines for specific time points is due to missing data.

In light of the results obtained from the desk research, it is evident that the organic sector is growing dynamically, becoming a part of a global economy. Europe, next to the USA, is the most important market for organic products, while the majority of the world’s 4.5 million organic farmers live in Asian and African countries (one-fifth of the world’s organic producers are found in Africa) [41]. Among the territories included in our SysOrg study, African Morocco is becoming an emerging organic producer (e.g., in organic citrus fruits), while in Europe, Denmark has the highest organic market share worldwide (12%), and Germany, after the USA, has the biggest organic market (15.3 billion EUR), becoming one of the three most important organic importers in the world [41]. However, the organic sector within Europe differs from country to country, and consequently, European case territories are found in different stages of organic transformation. Denmark and Germany are the leaders in the organic food system transition, followed by Italy, with 2.3 million hectares of organic farmland and the highest number of organic processors and producers in Europe [41]. Denmark has the highest organic consumption (365 EUR) per capita per year, followed by Germany (181 EUR), while in Italy, it is 62.2 EUR, and in Poland, it is only 8.2 EUR [41] (Figure 4).

To compare the status of organic consumption, it is important to understand the differences in the financial situations in each of the countries. The highest levels of income per inhabitant in the EU were recorded in the Central and Nordic EU Member States. Furthermore, the highest national median gross hourly earnings in the EU were 11 times higher than the lowest ones [42]. In 2018, the highest average gross hourly earnings in the EU were recorded in Denmark (27.20 EUR). For the other SysOrg’s European countries, they were 15.70 EUR in Germany, 12.61 EUR in Italy, and 4.98 EUR in Poland [43]. The percentage of the income spent on organic food in those countries in 2022 was highest in Denmark and lowest in Poland (Table 1), opposite to the percentage of household budget spent on food and non-alcoholic beverages (18.7% in Poland, 14.4% in Italy, 11.8% in Denmark, and 11.5% in Germany) [42], which confirms the trend that consumers in lower-income countries spend a greater proportion of their budgets on food than those in higher income countries [40]. Therefore, wealthy consumers can spend more money on food and buy more organic products (which tend to be more expensive than non-organic ones), and at the same time, allocate a smaller part of their HH budgets in comparison with more financially challenged consumers [44]. Another variable determining how much organic food is purchased by residents of SysOrg countries may be the food products prices, which differ considerably, e.g., the average monthly price of 100 kg of organic eggs in June 2024 was 523.32 EUR in Germany compared to 375.52 EUR in Poland, while for non-organic eggs, it was 205.11 EUR in Germany and 180.72 EUR in Poland [45,46].

**Figure 5:**
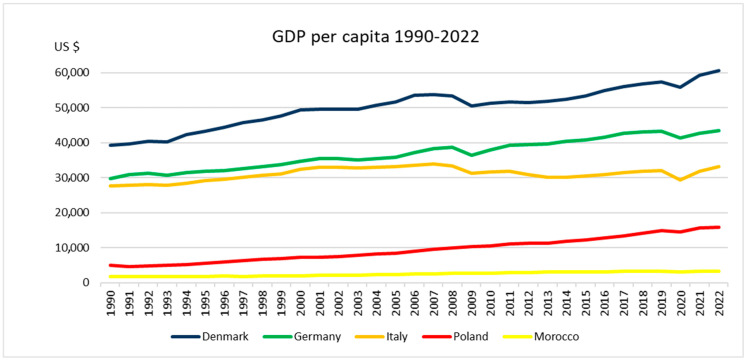
The growth of the GDP in USD per capita from 1990-2022 in five countries. Own elaboration based on the World Bank data.

To set the scene and better understand the border setting and conditions of the organic sector, interlinked with the economy of each European and African SysOrg country, we have compared the development of gross domestic product (GDP) in all of them. Compared to the European one, the African economy is in the developmental stage [47], although five African countries recorded a GDP per capita in 2016 higher than some of the EU members (e.g., Bulgaria) [48]. Morocco’s gross national income (GNI), converted to USD using the World Bank Atlas method, is lower than in the European countries included in this research: in Morocco, the GNI accounted for 3620; in Poland for 16,910; in Italy 36,420; in Germany 52,050; and Denmark 69,780 [49]. Akin is the growth of the GDP in USD per capita from 1990 [50] (Figure 5).

However, Morocco is one of the African leaders in ecological organic agriculture (EOA) practices, with 1.0 million hectares of agricultural land dedicated to EOA, and the country is hosting diverse international projects that are aimed at developing the organic sector [41].

**Figure 6:**
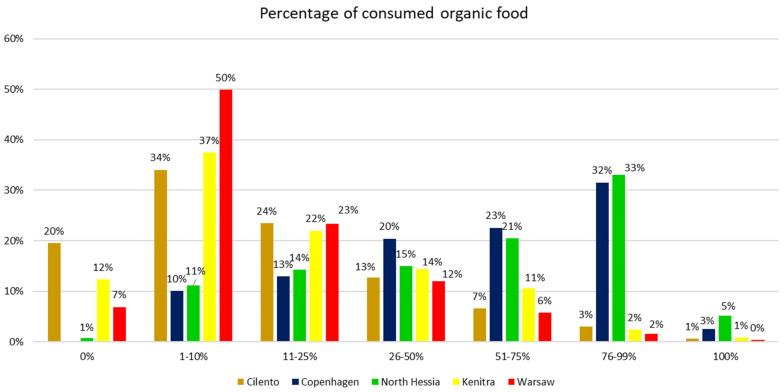
Answers to the household survey question “What percentage, by volume, of the foods you eat is organic?” by respondents from five case territories (%). Pearson’s chi-square test results for the overall relationship between variables (*p* < 0.05).

Zooming in on countries’ data to case territories, we can establish that there are statistical differences between the territories regarding the percentage of organic food consumed; however, it does not correspond exactly to the national data on organic consumption. The declared share (in %) of organic food in the diets of the respondents representing the five SysOrg case territories, based on the SysOrg HHS, differs significantly. In Copenhagen and North Hessia, the average declared share of organic food in the respondents’ diet was much higher compared to the remaining territories, which corresponds to the national data. Over 20% of the surveyed Copenhagen and North Hessia citizens declared a 51–75% share, and over 30% of the respondents had a 76–99% share, while 5% of the respondents in North Hessia and 2% in Copenhagen declared that 100% of the food they consume is organic. Moreover, in Copenhagen, not a single respondent declared not to consume organic food at all (Figure 6).

At the same time, in Warsaw, Kenitra, and Cilento, the majority of respondents declared much lower organic food shares, with 1–10% appearing as the most frequent response. There was a very low number of surveyed HHs in these territories who declared an organic food share of over 50%. The highest percentage of respondents who declared that they do not eat organic foods at all was noted in the bio-district of Cilento, followed by Kenitra and Warsaw (approx. 19%, 12%, and 5%, respectively).

**Figure 7:**
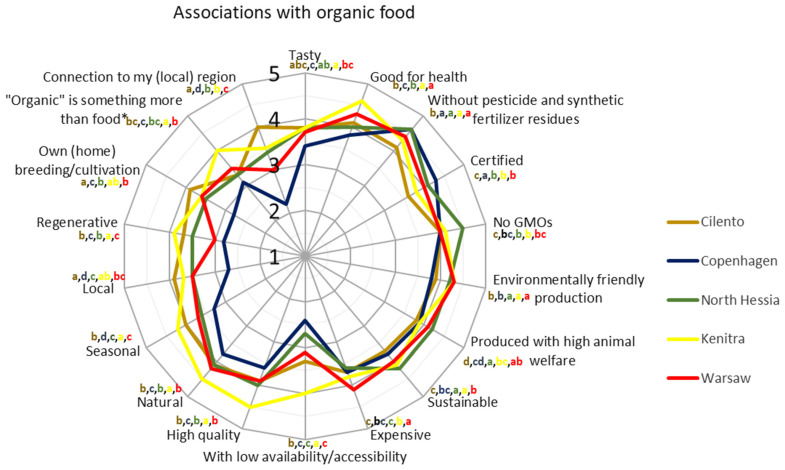
Answers to the household survey question “How much do you associate the characteristics listed below with organic food?” by respondents from five case territories. The lines represent the average values of gradings categorized from 1 (no association at all) to 5 (strong association). The different letters represent statistically significant differences between territories (Kruskal–Wallis rank-sum test, *p* < 0.05). * It is a lifestyle and philosophy; it transports values.

It is important to add that the consumers in the five SysOrg case territories associate different characteristics with organic food, which can be partially aligned with the above-mentioned organic and economic country-sector specifications and, in part, signals the root causes of the discrepancy between national and regional data (e.g., like influencing consumption), which is explained in the following chapters (Figure 7).

**Figure A1:**
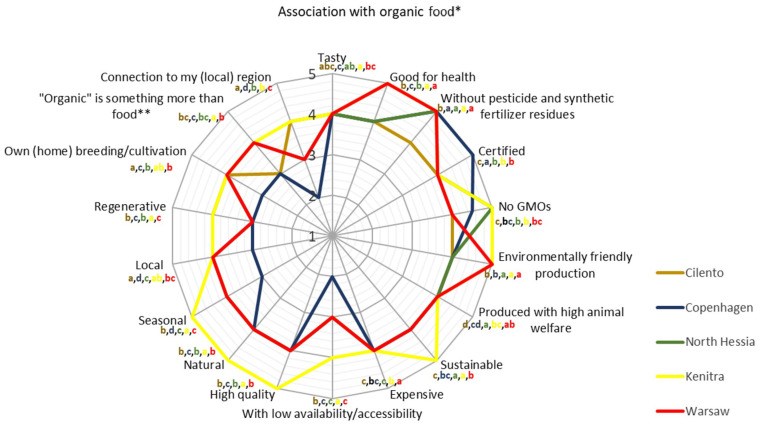
Answers to the household survey question “How much do you associate the characteristics listed with organic food?” by respondents from five case territories. * The lines represent the median values of gradings categorized from 1 (no association at all) to 5 (strong association). The different letters represent statistically significant differences between territories (*p* < 0.05). ** It is a lifestyle and philosophy; it transports values.

The expression “expensive” had a strong association with organic food for the surveyed Warsaw residents compared to the respondents from other territories. For the surveyed residents of Kenitra, it was significantly stronger compared to the North Hessian and Cilento respondents. For the Kenitra respondents, “low availability/accessibility” had a stronger association with organic food compared to the respondents from all other territories. For both the Copenhagen and North Hessian respondents, “low availability/accessibility” had the weakest association compared to all other territories. Characteristics such as “natural”, “seasonal”, and “high quality” were more strongly associated with “organic” for the Kenitra respondents compared to those from all other territories (Figure 7 and Figure A1).

On the other hand, “no genetically modified organisms (GMO)” and “produced with high animal welfare” were associated more strongly as organic descriptors for the North Hessia residents compared to the respondents from other territories. “Sustainable” was the strongest association for the North Hessian respondents and Kenitra ones, compared to others. “Certified”, as a descriptor of organic food, was stronger for the Copenhagen respondents compared to the respondents from other territories, while it appeared to be the weakest association for the Cilento sample. For the surveyed residents from Cilento, “connection to my (local) region” was more important as a descriptor of organic compared to all other territories and “local” compared to Copenhagen, North Hessia, and Warsaw. For the respondents from Copenhagen, both of those associations were the weakest. “Without pesticides and synthetic fertilizer residues” was the weakest association when thinking about organic food for the Cilento residents compared to the residents of all other territories (Figure 7 and Figure A1).

While the share of organic food in the total food market and consumption are important indicators of the development of the sector, it is also useful to consider the organic market positions that individual products can achieve. In many countries, organic eggs stand out in the retail market, often reaching impressive proportions of the entire egg market. For example, in Denmark, organic eggs have captured more than 30% of the egg market share in terms of value [41].

**Figure 8:**
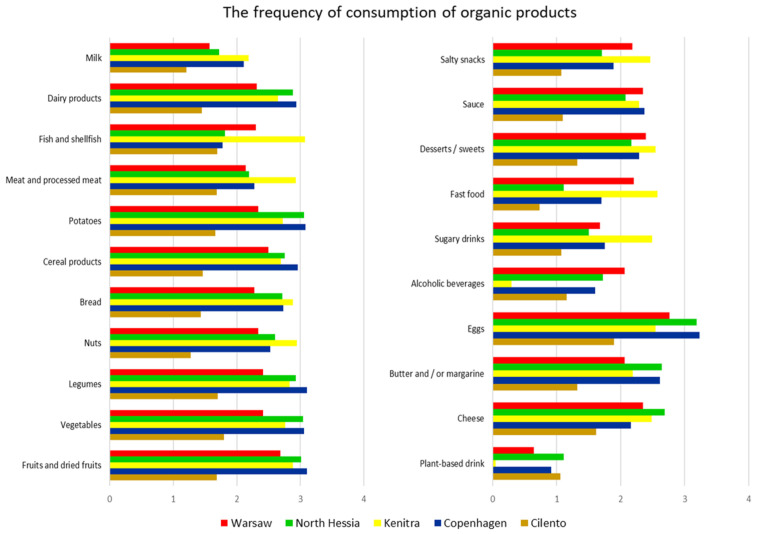
The frequency of consumption of organic products representing various food groups in the five case territories—results of the SysOrg household survey (based on the question: “How often are the [product group] you eat certified organic?”). The bars represent average values of frequencies that are categorized as 0—never, 1—rarely, 2—sometimes, 3—often, and 4—always. Pearson’s chi-square test results for the overall relationship between variables (*p* < 0.05).

The organic egg phenomenon and its popularity are also documented in our research, based on the HHS data, especially for Copenhagen, North Hessia, Warsaw, and Cilento (Figure 8), which corresponds to the national results found in the literature [50,51,52].

The market for organic vegetables and fruits is increasing globally, and while comparing product groups, it is attaining the highest market share of more than 10 percent in many countries [41]. According to the HHS, the above-mentioned food groups, together with potatoes and legumes, are also the most preferred organic choices compared to all other organic products mentioned in the survey (after eggs) for the respondents from Copenhagen, North Hessia, and Warsaw (in WA, cereals instead of potatoes). For Kenitra residents, organic fish and seafood were the most frequent choices (Figure 8), which might be connected to consumers’ misunderstanding/confusion about organic farmed versus wild-caught fish [53].

**Figure 9:**
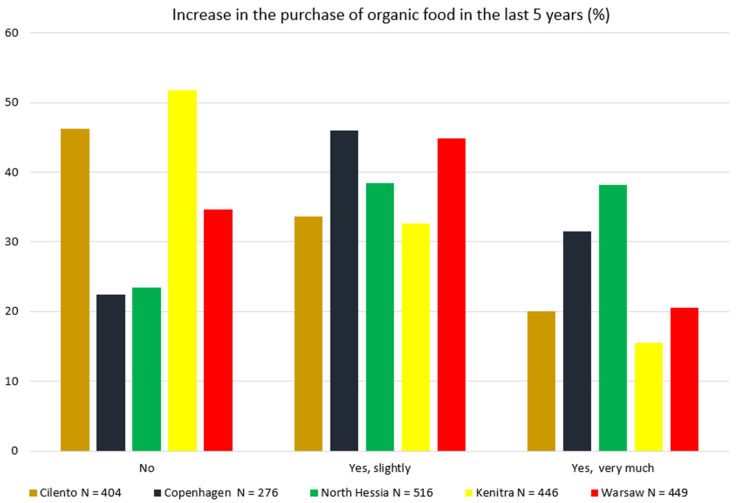
Answers to the household survey question “Has the purchase of organic food in your household increased in the last five years?” by respondents from 5 case territories. Pearson’s chi-square test results for the overall relationship between variables (*p* < 0.05).

The organic consumption at the HH level in the majority of SysOrg territories (except for Kenitra) is increasing. Most of the respondents in these territories declared that the purchase of organic foods in their HHS has increased in the last 5 years, either by very much (mainly Copenhagen and North Hessia) or at least slightly (other territories). The lack of such an increase in organic food purchases in recent years was indicated by only approximately 20% of North Hessian and Copenhagen citizens, but nearly 40% of the respondents from Cilento and Warsaw, and over 50% of the Moroccan survey participants (Figure 9). According to [40], in 2022, organic retail sales in Europe faced a first decline of 2.2% (2.8% in the European Union) since records began in 2000. Therefore, to further develop the organic sector globally, it would be important to better understand the country- and region-specific drivers and barriers.

### 3.2. Barriers and Drivers of the Organic Sector

#### 3.2.1. Barriers

**Figure 10:**
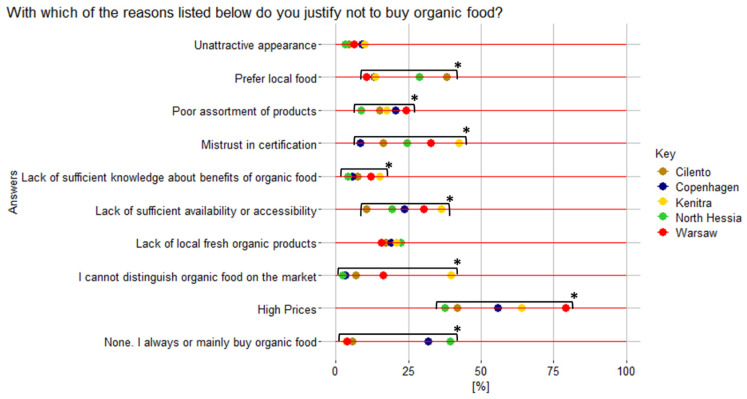
Reasons for not buying organic food declared by household survey respondents (%) from five case territories. * Statistically significant differences; Pearson’s chi-square test results for the overall relationship between variables (*p* < 0.05).

Based on HHS results, high price, mistrust in certification, and lack of sufficient availability/accessibility were three major reasons for not buying organic food (55%, 26%, and 24%, respectively), with all three being significantly more important for the residents of more financially challenged and less organically oriented territories: Kenitra (64%, 42%, and 36%, respectively) and Warsaw (79%, 33%, and 30%, respectively) (Figure 10). In fact, the price being a main barrier to buying organic products reappears in various Moroccan and Polish studies, such as the report about the Polish organic market by [54] or the comparison study of organic food consumption in African neighboring countries Morocco, Algeria, and Tunisia [55].

**Figure 11:**
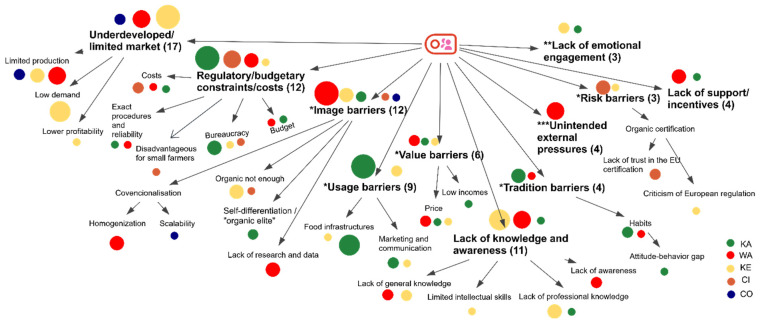
Hierarchy model of codes and subcodes displaying barriers of the organic food system in 5 case territories based on each of the initiatives’ individual analysis (SSI data). In brackets, no. of times mentioned. The size of the dots indicates the no. of times mentioned, while the colors indicate the territory. Deductive categories are based on the reviews by * Kushwah et al. (2019) [26], ** van Geffen et al. (2020) [30], and *** Béné et al., 2020 [31].

This also corresponds to the SSI’s results that reveal high prices of organic products and lower incomes in certain territories as significant value barriers and is especially noteworthy for the WA, KE, and KA informants (Figure 11).

*“Organic food is associated with a high price. So, it’s a challenge to make the supply chain shorter, fewer middlemen (…) who increase the price, the first barrier is price (…) that’s a key”*

(Warsaw)

Mistrust in certificates may also be connected to a higher price barrier. According to the data provided by the Polish research institute IMAS in 2021, Poles are quite familiar with the EU organic certificate; nonetheless, many of them do not trust the quality behind it, and consequently, they do not accept the higher price of organic products [54]. Additionally, the organic certification process, with its complex regulations, was criticized and reported to be mistrusted by some informants, constituting one of the “risk barriers” identified through the SSIs in rural territories (CI, KE) (Figure 11).

*“This is certification by a third party, which absolutely does not guarantee that the product is truly organic.”*

(Cilento)

*“The first thing that organic farming brings to mind is that it is a law with its advantages, but also its disadvantages.”*

(Kenitra)

Another obstacle that was uncovered in our qualitative study and connected to the “risk barriers”, however, which is more oriented toward farmers’ and producers’ work, are the “regulatory/budgetary constraints/costs”, which include both complicated procedures, their costs, budget limits, and a complicated bureaucracy related to organic certification. This is especially central for the informants from KA, who have stated perceived injustice between the organic and conventional sectors, and informants from CI, who claim insufficient benefits for their smaller, local farmers:

*“It comes with an enormous administrative workload. It comes with costs. You need to have a big intrinsic motivation, big idealism, to do so (…).”*

(North Hessia)

*“The negative aspects are what hinders the transition, such as the bureaucracy linked to organic farming, absurd (…).”*

(Cilento)

*“There are small farms, often of one hectare, two hectares, for which it would certainly not be worthwhile to activate the organic certification process and the costs would be considerably higher than the benefits.”*

(Cilento)

In Cilento, comparably with the other territories, preference for local food was amongst the most significant reasons for not buying organic food, according to the HHS (38% of respondents) (Figure 10). In fact, the establishment of bio-districts was often triggered by efforts to improve the marketing of regional products [56]. Therefore, there is a high fragmentation of small farmers, producing according to IFOAM schemes, that are trusted by the local community more than “third party” certificates.

*“In the Cilento bio-district, there are 10,000 organic producers. The problem is that only 1/10 of these probably have organic certification, the others produce organic according to IFOAM schemes, therefore also according to regenerative organic agriculture, and follow the latest world regulations (not EU’ organic) (…). The benefits for the environment are much more interesting than the marketing discourse.”*

(Cilento)

In fact, in Cilento, the local dimension is also an incentive on the financial supply side. Building relevant networks amongst farmers, entrepreneurs, municipalities, and consumers is a base of reciprocal trust. Moreover, decisions on “organic” criteria reflect the current reality and are based on assessments of the products by local stakeholders and their engagement [57].

The “undeveloped/limited market”, a barrier identified through the SSIs, is also connected to the aforementioned HHS results as follows: “lack of sufficient availability/accessibility” was indicated as a reason for not buying organic food by 36% and 30% of the Kenitra and Warsaw respondents, respectively, as well as a “poor assortment of products” being a more significant obstacle for respondents from the Polish capital city than the others (24% of respondents) (Figure 10) and, especially in Warsaw, it is connected to the SSI’s “limited production” sub-category of barriers (Figure 11).

*“Due to the small number of organic producers, we have limited access to the diversity of organic products and we have to attract people from all over.”*

(Warsaw)

In North Hessia, according to HHS results, a “lack of local fresh organic products” was indicated as a reason for preventing consumers from buying organic food by more respondents than in other territories (Figure 10)—an issue partially clarified by the SSIs within the “usage barriers”, both from the side of food infrastructure and marketing and communication (Figure 11).

*“We won’t manage to establish a purely local food system. If we still stick to the idea of a local system, we might not be able to make it. We are not big enough to supply this way.”*

(North Hessia)

Some research claims an uneven development of rural areas in Germany and problematic disparities in the state of Hessia [58]. The center of organic farming lies predominantly in the southern regions of the country, e.g., Bavaria, Baden-Wuerttemberg, Brandenburg, and Mecklenburg-Western Pomerania [59], with conditions facilitating conversion to organic; although Hessia has a high share of organic farming, reaching almost 17% [60]. Two states, Bavaria and Baden-Wuerttemberg, also recorded the highest purchasing power per capita in 2023 [61], while Hessia was in fourth place. Additionally, in our qualitative study, within “value barriers”, the sub-category “low income” was mentioned by the KA informants, stating that in North Hessia, the incomes are lower than in the other regions (Figure 11).

For respondents from Kenitra, compared to other territories, the most important barriers, according to the HHS, were “lack of capacity to distinguish organic food on the market” and “lack of sufficient knowledge about benefits of organic food (for health, environment, etc.)”, 40% and 15%, respectively (Figure 10). This is also confirmed and explained by the SSI’s results within the barrier of “lack of knowledge and awareness”, which was mentioned the most by Kenitra’s territory informants (Figure 11). This applies to farmers, producers, and consumers:

*“So, first of all, the barrier is the lack of knowledge of what the quality of an organic food is by the population.”*

(Kenitra)

Within this barrier, the qualitative investigation pointed out another issue:

*“The blocking factors are the training of farmers because they cannot manage projects that are not very simple, especially at the level that requires a certain intellectual level. That is to say, the farmer must respect a set of specifications, must fill out monitoring books to have a certificate and also must have intellectual skills to communicate and seek special consumers. It is not like conventional agriculture, in which the farmer sells his vegetables and products simply in a wholesale market. So, the organic farming project requires a certain organization compared to other projects.”*

(Kenitra)

In fact, according to the 2022 World Bank data, only 77% of the Moroccan population was literate (compared to Poland with 100% and Italy with 99%) [62]. In rural areas of Morocco, the percentage is much lower, especially among females, reaching as little as 10% [63].

Another set of barriers exposed by the SSIs and connected to “awareness and education” are “the image barriers”, consisting of perceived skepticism towards organics when compared with conventional foods, mostly due to unfavorable “promotion” and forced conventional standards of homogenization, which was especially underlined by the WA informants.

*“One thing that is very irritating and sad is the criticism of organic food from various sides. This criticism comes from conventional producers, from certain journalists, and from certain doctors.”*

(Warsaw)

Within this barrier, issues with the scalability of organic food were mentioned, mainly by the CO informants. On the other side, perceiving the organic sector as a hermitized “elite” sector was stated by the KA informants. Viewing organics as not sustainable enough was perceived by the KA and CI informants.

Other important barriers, reported mostly by the WA informants, were unintended external pressures (e.g., urbanization and the COVID-19 pandemic) and a lack of support and incentives.

A lack of emotional engagement from stakeholders in the developing organic sector was mentioned mostly by the KA informants, while habits and cognitive dissonance (classified as “tradition barriers” by Kushwah et al., 2019 [26]) enabled the former barrier, as was stated by the KE informants.

The HHS result “I always/mainly buy organic food” (Figure 10) was indicated mostly by the North Hessia and Copenhagen residents and rarely by the Kenitra and Warsaw ones, which confirms the previous discussion and SSI data.

#### 3.2.2. Drivers of Sustainable Organic Transition

**Figure 12:**
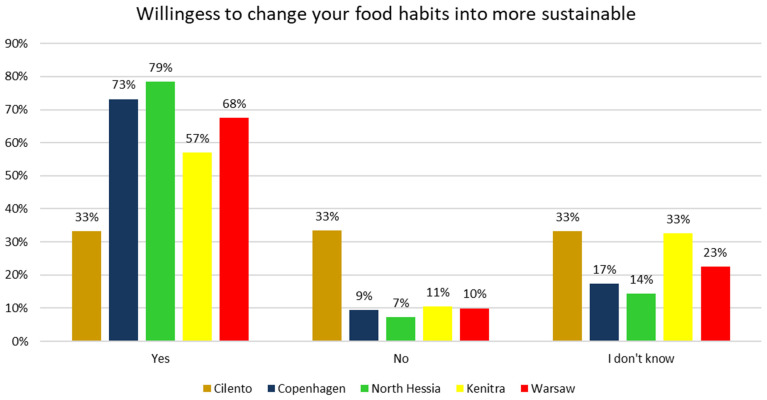
Answers to the household survey question “Would you like to change your food habits into more sustainable ones?” with % of respondents from five case territories. Pearson’s chi-square test results for the overall relationship between variables (*p* < 0.05).

**Figure 13:**
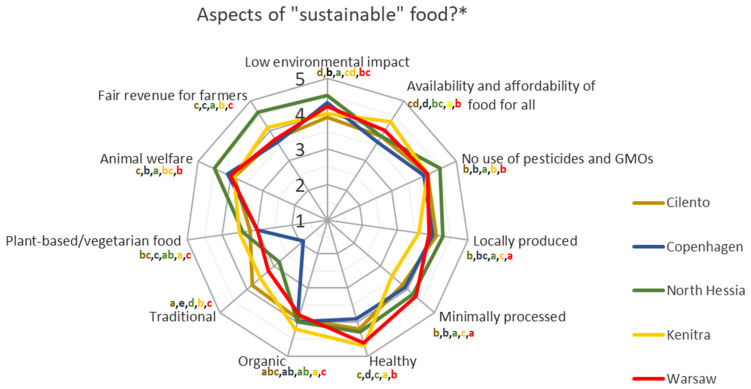
Associations with sustainable food for respondents from five case territories. * The lines represent the average values of gradings categorized from 1 (no association at all) to 5 (strong association). The different letters represent statistically significant differences between territories (*p* < 0.05, Kruskal–Wallis rank-sum test).

The majority of HHS respondents would like to change their dietary habits into more sustainable ones. The most willing to change are the respondents from North Hessia, contrary to the respondents from Cilento (Figure 12). It is worth mentioning that for the respondents from North Hessia, according to the HHS results, the most important aspects that they associate with “sustainable food” are those classified as social and environmental (fair revenue for farmers, animal welfare, and low environmental impact), while for the Cilento residents, it is healthy and locally produced food (Figure 13) which, based on previous research on this bio-district [56] is already widespread in this Italian territory. In both territories, the aspect of “no use of pesticides and genetically modified organisms (GMOs)”, which can be perceived both as an environmental and health-related characteristic, was highly associated with “sustainable food” (Figure 13).

The willingness to change food habits into more sustainable ones was also often reported by the surveyed consumers from Copenhagen and Warsaw but less by the respondents from Kenitra (Figure 12); however, in KE, the “organic” aspect was more important when thinking about “sustainable food” than in the other SysOrg territories (Figure 13).

**Figure 14:**
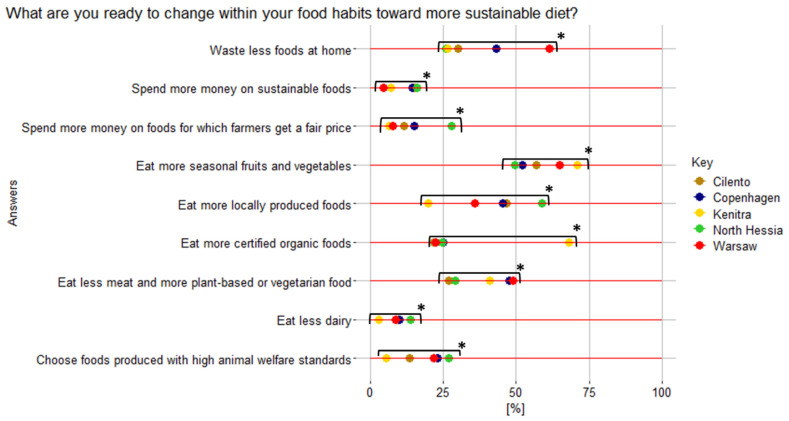
Declared actions that HHS respondents from five case territories are ready to undertake to change their food habits into more sustainable ones. * Represents statistically significant differences; Pearson’s chi-square test results for the overall relationship between variables (*p* < 0.05).

**Figure A2:**
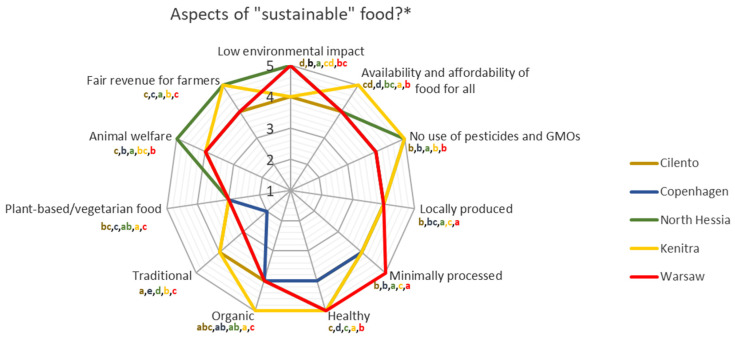
Associations with sustainable food for respondents from five case territories. * The lines represent the median values of gradings categorized from 1 (no association at all) to 5 (strong association). The different letters represent statistically significant differences between territories (*p* < 0.05). Differences between independent multiple groups for variables on the ordinal scale were assessed using the Kruskal–Wallis rank-sum test.

For the respondents from the five case territories who have answered “yes” to the question “Would you like to change your food habits into more sustainable ones?” (Figure 12 and Figure A2), the top three actions they indicated as most possible to undertake were as follows: eating more seasonal fruits and vegetables (58.5%), locally produced foods (42%), and less meat and more plant-based/vegetarian food (39%) (Figure 14). Therefore, to increase interest in organic food, all these characteristics should be considered, especially for territories such as WA, CO, and KE, where consumers showed a high willingness to change to more plant-based diets (49%, 47%, and 41%, respectively). This calls for increasing the share of organic plant-based food to reduce pesticide-induced health risks [56].

Readiness to change food habits into eating more certified organic foods was more often indicated by the respondents from Kenitra (68%), North Hessia, and Copenhagen (both 25%) compared to the other territories. For the KA and KE residents, compared to others, “organic” was also a more important association when thinking about “sustainable food” (Figure 13).

The importance of organic aspects in sustainable diets and the highly rated association of organics and sustainability in Kenitra may be a result of a close link between ‘Beldi’ products and organic ones. In Morocco, ‘Beldi’ products (an Arabic term referring to everything traditional that is nationally or locally produced) are often appreciated, and there is a positive association between organic and ‘Beldi’ foods [64]. However, this sometimes creates confusion and should be precisely explained, taking into consideration the health and nutritional aspects of organic products especially, which, according to research, are most attractive to Moroccan consumers [65].

Residents from territories with the highest earnings, KA and CO, also indicated spending more money on sustainable foods as a change that they would be ready to adopt (28% and 15%, respectively) more than others. Spending more money on foods for which farmers receive a fair price was the least feasible for residents from rural regions of KE (6%), CI (11%), and WA (8%). Akin to this, choosing foods produced with high animal welfare standards were KE (5%) and CI (13%) (Figure 14).

This indicates that egoistic drivers of sustainable change, like health, taste, and fewer residues, versus altruistic ones, like environmental protection, animal welfare, and social equality [66], might be based on the country-specific differences related to cultural and economic aspects and food security standards. This moderates the influence of either environmentally or health-oriented considerations on purchases of sustainable or organic products [67].

At the same time, social norms influenced by one’s social environment might be a decisive factor for actions and behaviors [68], which could be the reason why altruistic drivers are more important in organic-advanced food systems [69,70].

**Figure 15:**
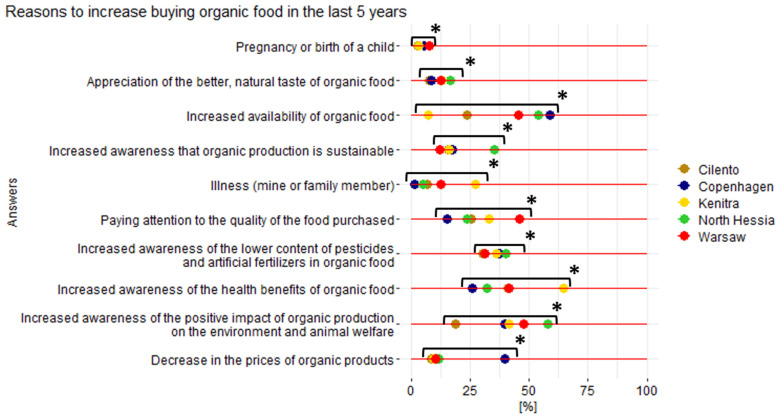
Declared reasons of the household survey respondents from five case territories to increase the purchase of organic foods in their households in the last 5 years. * Represents statistically significant differences; Pearson’s chi-square test results for the overall relationship between variables (*p* < 0.05).

In our study, for the residents of the five case territories who increased their purchasing of organic foodstuffs in their household in the last five years, the most important reported reason for doing so was, in general, increasing awareness of the positive impact of organic food/production on the environment, animal welfare, and health, and increased availability of organic food (Figure 15).

The increased awareness about the positive impact of organic production on the environment and animal welfare was the most important driver for all case territories, in general (44% of respondents), and especially for the KA respondents (58%). At the same time, the awareness that organic food is better for health (in general, indicated by 40%) was most important for Kenitra’s respondents (65%). This partially confirms that environmental concerns, as the top significant motives to purchase organic food, are found mostly in the studies in a developed nations’ context [71,72,73], while the personal drivers connected to health are more important in developing markets [74]. However, the increased knowledge about the lower amount of pesticide residues, artificial fertilizers, and food additives in organic food was indicated as an important reason for the increase in organic food purchases by 40%, 37%, and 36% of the residents from KA, CO, and WA, respectively, which is in line with previous national research [75,76].

The importance of raising awareness to change behavior toward the desired direction by using informative instruments (learning/persuasion) [77,78] is also supported by our qualitative research, “awareness and education”, which was the most mentioned driver revealed in four out of five case territories’ initiatives (Figure 15). This is connected to raising awareness by proper communication and educating the general public, as well as professional training for all stakeholders (chefs, managers, farmers, etc.).

*“In Warsaw, the most important thing is the dissemination of the values of organic farming and its products, (…) information path, because everyone will make their own decisions.”*

(Warsaw)

*“We would also need some kind of educational campaign, which would explain why we need organic farming, why this food is more beneficial.”*

(Warsaw)

*“The critical point is informing the population. It is essential, first of all to raise awareness.”*

(Kenitra)

*“First train the managers who will produce organic, i.e., convince the farmers around of the importance of this organic initiative.”*

(Kenitra)

*“Principles that we also use in our education of kitchen staff.”*

(Copenhagen)

Furthermore, the personally perceived health attributes of organic food, “devoid of harmful ingredients”, as well as the “quality and sensory aspects”, constituting drivers classified as “functional” (benefits derived from the functional features), were particularly reported by informants from the North Hessia, Cilento, and Kenitra initiatives, e.g.,:

*“The main motivation is wanting to eat well, therefore selfish (laughter), especially because at the beginning it was difficult to find organic products in the area where I live and therefore we started an association.”*

(Cilento)

*“What motivates me is to bring quality food to as many people as possible”*

(North Hessia)

Returning to the availability of organic food, which was the second most significant motive for increasing purchases of organic food in the last five years according to the results of the HHS (40% of respondents, in general), it was decisively more important for the Copenhagen and North Hessian residents (59%, 44%, respectively), while for the Moroccans, it was not so important (7%) (Figure 15). These territory-based differences may be connected to the development of the organic sector in each of the countries, described in chapter 4.1., and KA and CO being locations for highly growing organic markets and demand [78,79].

**Figure 16:**
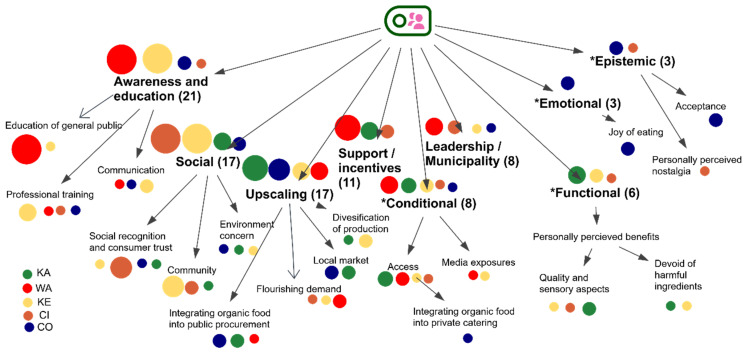
Hierarchy model of codes and subcodes displaying drivers of the organic food system in 5 case territories based on each of the initiatives’ individual analysis (SSI data). In brackets, no. of times mentioned. * Deductive categories based on the review by Kushwah et al. (2019) [26].

Moreover, several important “upscaling” drivers were identified through the SSI investigation, including increasing local markets (CO and KA), diversification of production (KE and KA), and integrating organic food into public procurement (WA, CO, and KA) (Figure 16).

*“It is obvious that if children are fed organically at school, or patients in hospitals, or people working in public institutions, then this can spread and this change will be socially understood and accepted, but it has to start from the top.”*

(Warsaw)

*“Conditional” drivers, on the other hand, depend on situations and circumstances like “access”—e.g., the vicinity to market (KE), privileged access to markets stand by organic farmers (CI), access to organic products (WA), and “media exposure”*

(KE and WA):

*“It is not only good to have a flashy spot, but also good information which goes through the TV channel.”*

(Warsaw)

*“I believe that there are opportunities, we are very close to the market, that is to say to the big cities of Morocco, that it is one of the things that can encourage organic agriculture or agroecology in our region.”*

(Kenitra)

The previous research situates a proactive approach to keeping good health due to certain conditions [26], which is also under the “conditional” category, which is connected to our quantitative HHS results, such as the motivation of “paying attention to the quality of the food bought for the family” for increasing organic food purchases was especially reported by the Warsaw residents (46%), and “due to illness” was reported by the respondents from Kenitra (27%).

The joy of eating was mentioned among important organic sector drivers (classified as “emotional drivers”) by the CO informants, while acceptance and familiarity with organic food (classified as “epistemic drivers”) were mentioned by the informants representing the interviewed CO and CI initiatives.

### 3.3. Entry Points

**Figure 17:**
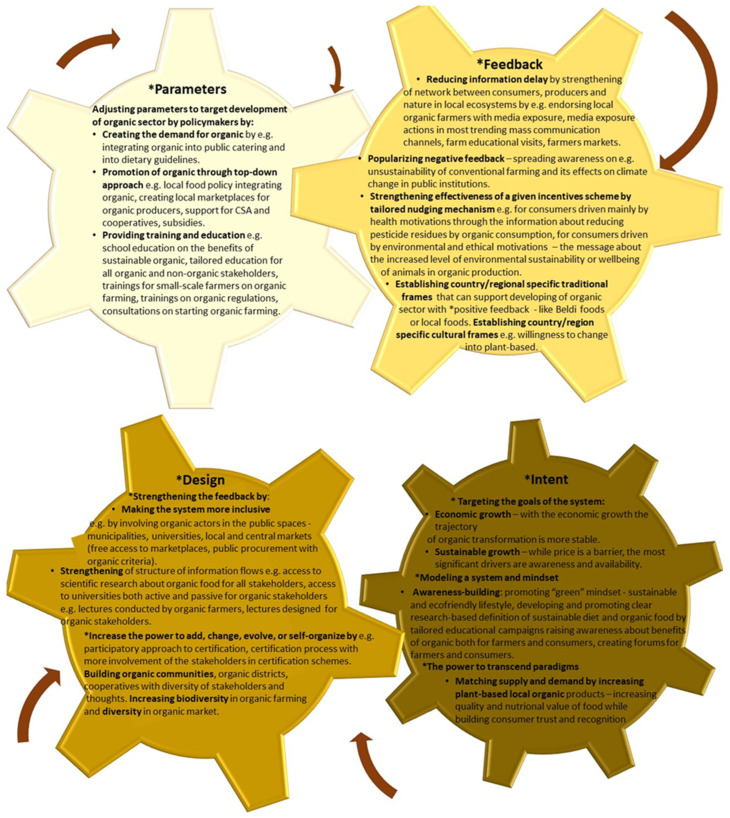
Proposed types of interventions (entry points) in different system characteristics (*) (realms) based on own research in five case territories of the SysOrg project. Arrows demonstrate a possible interaction between those realms—a small shift in one thing can produce big changes in everything.

This study has established that the territories are at a diverse stage of organic development; however, both the qualitative and quantitative results demonstrate the complex interplay between the drivers and barriers influencing organic food consumption and production in each territory. This interaction has allowed us to extract potential entry points based on Donella Meadows’ concept of leverage points in the complex system, concentrating on the four following realms: parameters (modifiable, mechanistic characteristics; target by policymakers), feedback (the interactions between elements within a system of interest), design (related to the structure of information flow, rules, power, and self-organization), and intent (characteristics related to the norms, values, and goals embodied within the system of interest and the underpinning paradigms out of which they arise), where a small shift in one thing can produce big changes in everything. Based on Abson et al. (2017) [29] and the interpretation of Meadows’ theory and the diverse level of organic development in the described territories, we have concluded that the outcomes of interventions through entry points would have different levels of effectiveness towards organic transformation with varying interchange between individual realms (Figure 17).

## 4. Conclusions

This paper has broadly characterized drivers and barriers towards the development of sustainable organic food systems in five case territories, both from the perspective of HHs and the selected initiatives. This research demonstrates that an understanding of sustainable organic food systems varies among the studied territories, e.g., the community-led organic food system in the bio-district Cilento vis-à-vis the certified organic-oriented public food system in Copenhagen. However, sustainable changes regarding food habits, e.g., increasing seasonal fruits and vegetables or plant-based/vegetarian local food, were chosen as the preferable options in all territories collectively (especially for CO and WA) and, therefore, should be included in food policies influencing the growth of an organic, sustainable food sector. This study has also revealed that economic, regional, and cultural conditions may influence the motivations and obstacles to sustainable organic food system development. We also observed differences between the barriers faced by all surveyed consumers and the drivers for those who have increased their organic intake in the last five years. While the price may be the biggest barrier, increasing awareness about the benefits of organic food and organic food availability are the biggest drivers for organic consumers, rather than a lower price.

The approach used in this study has its strengths but also limitations. The most significant limitation was the use of the “river” sampling methodology, a non-probabilistic assessment, which, however, permitted us to reach many participants in a limited time. The biggest strength of this study is the use of a mixed methods approach, which allowed us not only to enrich data but also to collect it from citizens using quantitative methods and to target transitional initiatives using a qualitative approach, catching similarities and differences while apprehending a more comprehensive representation of the dynamics of the different food systems and extracting entry points.

We believe that the diversity of territories, stakeholders, and perspectives in this study shows the complex interplay between the drivers and barriers influencing sustainable food consumption and production and, thus, can enrich research on the sustainable organic food and public health sectors. The entry points identified through this method exhibit varying degrees of interchange among distinct realms; however, they are universally applicable and grounded in practical solutions. These solutions have the potential to enhance political and educational initiatives aimed at the transformation of sustainable food systems. Future research should expand the utilization of the HHS and SSI to a larger and more representative sample, and investigate data from territories outside the European Union as well as document presented entry points.

## Figures and Tables

**Table 1:** The percentage of income spent on organic food in European countries included in SysOrg project calculated based on annual gross revenue and expenditure on organic food per person per year in 2022 [41,43].

Case Territory’s Country	Annual Gross Revenue (EUR) (per Person/Year)	Percentage of Income Spent on Organic Food (per Person/Year)
Denmark	40,436.73	0.90%
Germany	35,740.18	0.51%
Italy	23,286.80	0.27%
Poland	12,503.72	0.07%
Morocco	*n.d.	*n.d.

* n.d.—missing data.

## Data Availability

The original contributions presented in this study are included in the article/Supplementary Materials. Further inquiries can be directed to the corresponding author.

## Supplementary Materials

The following supporting information can be downloaded at: https://www.mdpi.com/article/10.3390/nu17030445/s1, Table S1: Household survey—selected questions used for this publication, Table S2: Proposed types of interventions (entry points), Figure S1: Research design employed in the present study, Figure S2: Process of initiative selection.

## Abbreviations

The following abbreviations are used in this manuscript:

CBE	All consumption-based emissions
FAO	The Food and Agriculture Organization of the United Nations
FS	Food system
GHG	Greenhouse gas
GMOs	Genetically modified organisms
HH	Household
HHs	Households
HHS	Household Survey
NDGs	National dietary guidelines
O	Organic perspective
OECD	Organization for Economic Co-operation and Development
SFS	Sustainable food system
SSI	Semi-structured interview
W	Waste perspective
WHO	The World Health Organization
CI	Cilento in Italy
CO	Copenhagen in Denmark
KA	North Hessia in Germany
KE	Kenitra in Morocco
WA	Warsaw in Poland

## Appendix A

**Table A1:** Description of five case territories (based on desk research).

Territory:	Warsaw	Cilento Bio-District	Copenhagen	North Hessia	Kenitra
Code:	(WA)	(CI)	(CO)	(KA)	(KE)
	SGGW *	CREA * CIHEAM *	UCPH *	UKA *	UIT *
	The capital city of Warsaw is located in east-central Poland in the Voivodeship Masovia, with a total area of 517.2 km^2^. The SysOrg territory corresponds to Warsaw City inhabited by over 1.86 million people and divided into 18 districts. The city of Warsaw is now working on the food policy of the city.	Cilento bio-district (not an administrative state) is located in the Campania region in Italy, in the National Park of Cilento, Vallo di Diano, and Alburni. It covers 3196 km^2^, has a population of 270,000 inhabitants, and includes 40 municipalities and 400 organic farms. It is the first bio-district in Europe, a participatory and inclusive community where farmers, consumers, public authorities, and other local actors realize an agreement aimed at the sustainable management of local resources.	The capital of Denmark. The municipality of Copenhagen constitutes the historical city center with a population of 626,350 inhabitants and covers 86 km^2^. In 2009, the municipal council decided that Copenhagen would become the world’s first carbon-neutral capital by 2025; in 2014, it was a European green capital and aims to become the capital of sustainable development.	North Hessia (not an administrative state) is part of the state Hesse in west-central Germany. North Hessia covers 6908 km^2^ and has a a population of 977,622 inhabitants. The region is well known for organic agriculture since the world’s first chair for Alternative Agriculture was established there in 1981 (today, Organic Agriculture). In the case of North Hessia the HHS covered mainly the Werra-Meissner district.	Located in northwest Morocco, the Kenitra province is divided into five districts: three urban and two rural communes. The Kenitra province has 1,061,435 inhabitants and covers 112 km^2^. It has an agricultural area of 285,259 ha of which 70,769 ha are irrigated, making it important to regional production. Kenitra province is located in the region of Rabat Salé Kénitra.

* Scientific partners responsible for territories: SGGW—Warsaw University of Life Sciences (PL); CREA—Council for Agricultural Research and Economics (IT); CIHEAM-Bari—International Center for Advanced Mediterranean Agronomic Studies—Mediterranean Agronomic Institute of Bari (IT); UCPH—University of Copenhagen (DK); UKA—University of Kassel (DE); UIT—Ibn Tofail University (MAR). Additionally, FH Münster University of Applied Sciences (DE) was responsible for the transition perspective but not for a territory.

## Appendix B

**Table A2:** Questions were used in semi-structured interviews conducted in five case territories with representatives of organic-oriented initiatives. Questions were translated into each of the territory’s original languages. Interviews were recorded, transcribed, and translated into English again.

What does organic mean for you, personally? What do you associate with organic? Is it just a land use system/production method for you or something more?
What are your main motivations for undertaking the initiative(s) towards increasing the share of organic foods in your region? What drives you?
What are the principles by which you define an ideal local food system? (what is your vision of ideal local, sustainable food system?)
What is the range of organic food products produced in/around your territory (locally)? Is there vegetable production, or mostly dairy or other?
Do you recognize a presence of complete value chains of organic foods (farm to table) in your territory?
Are there any Food Hubs or other aggregation platforms and cooperative marketing in your territory, for making local organic food available to consumers at consistent quantities? Do you know about any efforts taken/planned to be taken to create an intentional local organic foodshed within your territory)?
Are there any knowledge transfer (training or apprenticeship)/educational programs, (regular) conferences etc. in organic food and farming in your territory?
Are there organic production systems such as e.g., organic urban farming, market gardening, diversified crop and livestock operations, CSAs (community supported agriculture), permaculture, agroforestry, food forests, no-dig etc. present in your territory? Please share/give best practice examples.
What are in your opinion the most important drivers and barriers of transition of your territory towards organic food system, with a larger share of organic foods? What are in your opinion the critical points for increasing the share of organically produced/consumed food in your territory?

## Appendix C

**Table A3:** Characteristics of the qualitative study sample: organic initiatives selected for semi-structured interviews (SSI) in five case territories.

Territory *	Founding Year/	Type and Legal Organization	Organic Perspective Rationale
WA	2019	Municipality	Functional and sustainable food policy, indorsing sustainable nutrition, good quality, and affordable food for all residents, and green public procurement (GPP) criteria promoting organic food in all public procurement.
WA	2010	Limited liability company	Organic food market, promoting organic food, healthy cooking, and lifestyle, focusing on organic products and supporting organic farming.
WA	2013	Registered association	Supporting local and organic farmers, producers, processors, and the local society. Spreading the initiative of responsible food shopping and providing access to organic products from small organic farmers to urban residents.
WA	2017	Sectoral organization	It is a sector organization of farmers, producers, processors, and sellers of certified organic food that promotes a sustainable lifestyle and broadly educates the public about the health benefits of organic products, farming, and processing.
CO	1999	Company	Boxing scheme with seasonal organic products and recipes written for meals.
CO	2001	Municipality/Company	An initiative promoting sustainability in public kitchens, training all actors in the public sector about sustainable and organic food, and helping to reach the goal of 98% organic food in public catering of the city.
KA	2014	Registered association	Promotion of collaboration and dialogue between different stakeholders active in the organic sector.
KA	2020	National Program	Increasing the area under organic cultivation and thus also connecting regional economic development and ecological production with its positive effects on the environment, i.e., biodiversity, climate, and soil.
KA	2016	Company	An organic restaurant with a small market supporting local, organic food production.
CI	2004	Organizational entity of the Eco-Region	A non-profit organization committed to organic transition, sustainable farming practices, sustainable tourism, gastronomy, social agriculture, education, etc.
CI	1976	Organic Cooperative	An association of organic farmers and experts in agronomy.
KE	2015	Registered association	Network of agroecological initiatives in Morocco that allow collaboration between all actors, and supermarkets that offer organic and local products.
KE	1998	Company	A co-op that produces organic honey, jams, argan, and olive oil with rural women, peasants, and regional producers, respecting fair trade, etc.
KE	2017	Registered association	An association of farmers who produce citrus, pomegranate, nectarine, almonds, figs, wheat, corn, sunflower, beans, watermelons, and pumpkins.
KE	2013	Registered association	An association of farmers who produce mostly fruits and vegetables.

* (WA) Warsaw; (CO) Copenhagen; (KA) North Hessia; (CI) Cilento; (KE) Kenitra.

**Table A4:** The characteristics of the household survey (HHS) respondents: gender, age, level of education, and disposable HH income; total sample, n = 2189.

Variables	%
Gender	
Female	57.1
Male	42.9
Age in years	
25 or less	13.8
26–35	23.4
36–45	19.8
46–55	17.5
56–65	13.8
66 and more	11.7
Level of education	
No formal/primary education	4.8
Lower secondary education	13.3
Upper secondary education	18.3
Apprenticeship	6.8
Bachelor’s degree or equivalent level	19.7
Master’s degree or equivalent level	31.1
Doctoral studies and/or higher	6.0
Disposable Net Household Income per year	
I prefer not to answer	28.2
Lower	13.8
Low	14.0
Medium-low	10.1
Medium	7.3
Medium-high	7.8
High	6.8
Highest	12.0
