# Supplementary material for: Entry Points, Barriers, and Drivers of Transformation Toward Sustainable Organic Food Systems in Five Case Territories in Europe and North Africa
Source: Nutrients. 2025 Jan 25;17(3):445. doi: 10.3390/nu17030445 (PMC11820227; doi:10.3390/nu17030445)
Supplement: Supplementary file 1 [file nutrients-17-00445-s001.zip › SM_Figures S1 and S2.pdf]

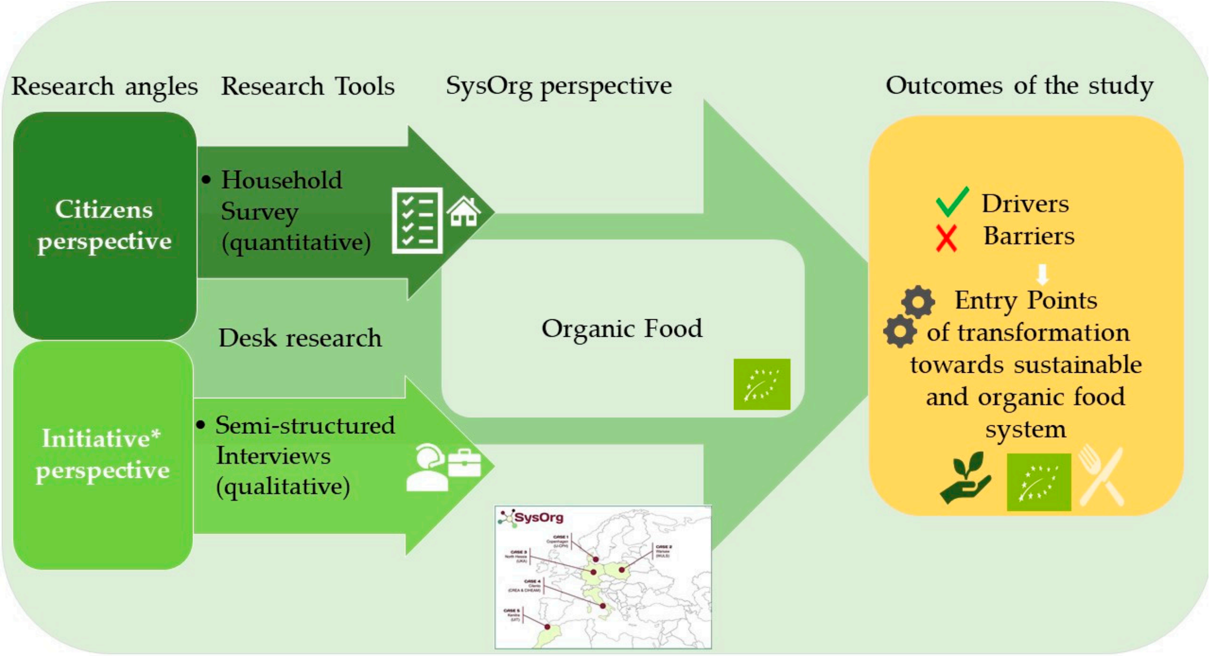

Figure S1. Research design employed in the present study.

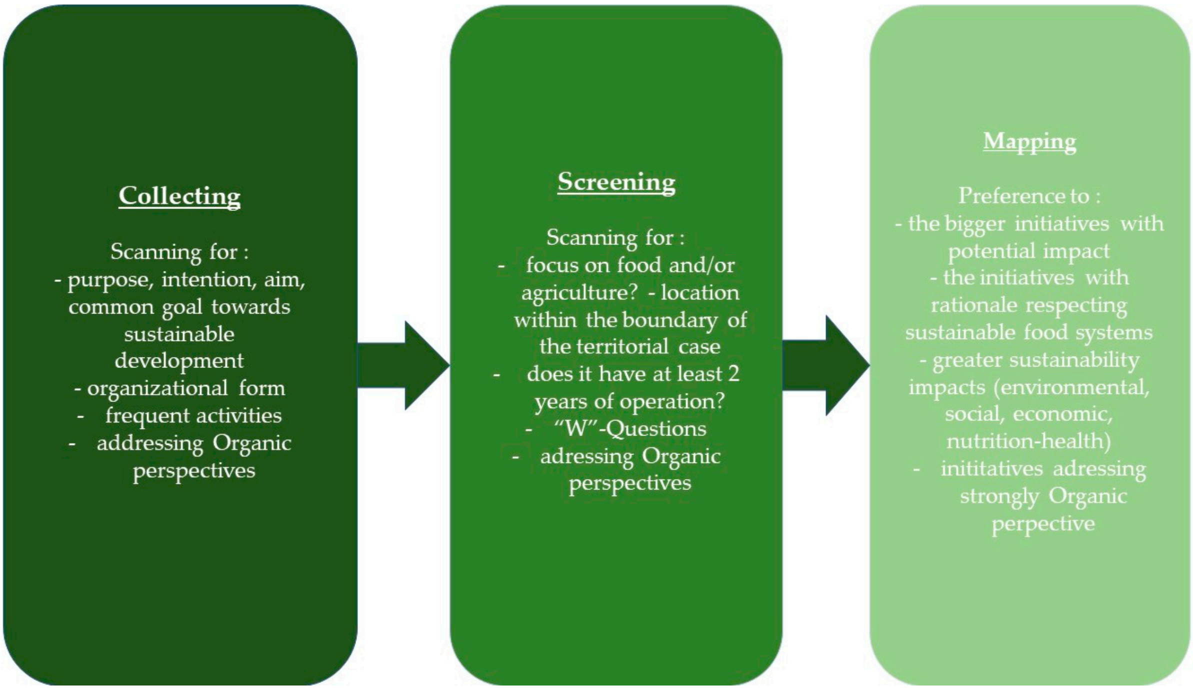

Figure S2. Process of initiative selection.
