# Supplementary material for: Entry Points, Barriers, and Drivers of Transformation Toward Sustainable Organic Food Systems in Five Case Territories in Europe and North Africa
Source: Nutrients. 2025 Jan 25;17(3):445. doi: 10.3390/nu17030445 (PMC11820227; doi:10.3390/nu17030445)
Supplement: Supplementary file 1 [file nutrients-17-00445-s001.zip › SM_Table S1.pdf]

Table S1. Household Survey – selected questions used for this publication

Household Survey Selected questions used for this publication
SysOrg CORE organic
Organic agro-food systems as models for sustainable food systems in Europe and Northern Africa
SUSFOOD2 Online Title: Household survey of diet, organic food consumption and food waste

Consent form
You are being invited to participate in a research study entitled "*Organic agro- food systems as models for sustainable food systems in Europe and Northern Africa*" (SysOrg) that is being conducted by [NAME of steering committee member], [POSITION, e.g. Professor] at [Institution]. This is a collaborative research with partners in Denmark, Italy, Poland, Germany and Morocco.<br><br>The questionnaire should take around 30 minutes to complete. Your participation in this study is voluntary. At any time and for any reason, you can refuse to answer a question or stop filling out the questionnaire.<br><br>If you agree to participate, you will remain completely anonymous, which means that none of the researchers who work with the analysis of the responses can identify who you are or have access to your personal data.The type of data assessed in this survey does not contain any personal information such as your name and is therefore not subject to the European General Data Protection Regulation (GDPR). The answers will be used exclusively for statistical purposes in compliance with current national legislation. The European project partners - University of Copenhagen, (DK), Warsaw University of Life Sciences, (PL), Council for agricultural research and economics - CREA, (IT), FH Münster University of Applied Sciences, University of Kassel (DE), and International Centre for Advanced Mediterranean Agronomic Studies – Mediterranean Agronomic Institute of Bari (CIHEAM-Bari), (IT) - will process the results of this study. The data collected will be used for survey reports and for scientific publications.<br><br>If you have any questions about this research, you can contact us by sending an email to [NAME].<br><br>**Consent**<br>The study described above has been explained to me. I understand that future questions I may have about the research will be answered promptly by the investigators listed above.<br><br>By selecting "next", the subject certifies that he/she is at least 18 years of age.<br><br>☐Yes, I agree to participate in this study<br>☐No*, I do not agree to participate in this study

Sociodemographic information			
**The questionnaire should be filled out by the person primarily responsible for preparing and purchasing food in your household.**			
**Do you live in this territory [TERRITORY NAME]?**			
☐Yes		☐No*	
**Which municipality/administrative part of the city/region (district) do you live in?**			
Name: ______________________________ Zip Code: _________________________________			
**Your age (in years)**			
**Your gender****			
☐Female	☐Male	☐No pronoun	☐ I prefer not to answer
**Your level of education****			
☐No formal education	☐Primary education (1-4 years)	☐ Lower secondary education (5-10 years)	☐ Upper secondary education (10-13 years)
☐Apprenticeship (2-3 years)	☐Bachelor's degree or equivalent level (3 years)	☐Master's degree or equivalent level (e.g.	☐ Doctoral studies (PhD) and/or higher

		Diploma) (3+2 years)	
**Household members**			
Total number of household members			
Age in Years	Female Members	Male Members	Other
< 1			
1-9			
10-17			
Adults (≥18)			
**Disposable Net Household Income (in Euro) per year****			
☐Up to 18.000	☐18.001-27.000	☐27.001-36.000	☐36.001-46.000 [choose average income of Territory]
☐46.001-57.000	57.001-72.000	☐More than 72.000	☐I prefer not to answer
**How much of your net monthly household income is approximately spent on food purchase per month?**			
☐<10%	☐10-25%	☐26-50%	☐>50%

**What percentage, by volume, of the foods you eat is ORGANIC?**<br><br>**ORGANIC food - produced according to standards for organic farming, ie. without synthetic pesticides, synthetic fertilizers, genetically modified organisms (GMOs), synthetic additives, and with the least possible processing (e.g. no radiation); organic production is controlled and products are certified by control bodies;**<br>**in the EU organic food products are labelled with "Euro-leaf".**						
☐0%	☐1-10%	☐11-25%	☐26-50%	☐51-75%	☐76-99%	☐100%

*cut-off, the survey ends here

**need to be adapted to case territory. NOT change the amount of answers due to comparability (*income: determine the average income (middle), place three lower and three higher categories*)

**food examples can be adapted to case territory if necessary due to eating culture/religion

***if the related food group was selected to be "never" eaten, this question will not show up

How important are the attributes listed below for your food choices? Please, rate. *(grading from 1 – not important at all, to 5 – very important).*

**Attributes**	**Grading**
Price	
Taste	
Appearance	
Safety (e.g. pathogens, pesticide residue)	
Sustainable packaging (e.g. biodegradable, reusable, non-plastic)	
Composition (ingredients)	
Nutritional value	
Origin (I prefer local products)	
Without genetically modified organisms (GMOs)	
Certificates	
Seasonality	
Freshness	
Naturalness (no artificial food additives)	

How important are the aspects listed below when you think about "sustainable" food? Please, rate. (*grading from 1 – not important at all, to 5 – very important*).

Low environmental impact	
Availability and affordability of food for all	
No use of pesticides and genetically modified organisms (GMOs)	
Locally produced	
Minimally processed	
Healthy	
Organic	
Traditional	
Plant-based/vegetarian food	
Animal welfare	
Fair revenue for farmers	

Would you like to change your food habits into more sustainable diets?

☐Yes	☐No	☐I don't know		
If yes, **what are you ready to change?***(Tick maximum 3 boxes)*				
☐Eat more certified organic foods	☐Eat more seasonal fruits and vegetables	☐Eat less meat and more plant-based/vegetarian food	☐Eat more locally produced foods	☐Eat less dairy
☐Spend more money on foods for which farmers get a fair price	☐Waste less foods at home	☐Choose foods produced with high animal welfare standards	☐Spend more money on sustainable foods	☐Other:_______________________

How much do you associate the characteristics listed below to organic food? (*Add gradings from 1 (no association at all) to 5 (strong association)*)

Tasty	
Good for health	
Without pesticide and synthetic fertilizer residues	
Certified	
No genetically modified organisms (GMO)	
Environmentally-friendly production	
Produced with high animal welfare	
Sustainable	
Expensive	
With low availability/accessibility	
High quality	

Natural	
Seasonal	
Local	
Regenerative	
Own (home) breeding/cultivation	
"Organic" is something more than food – it is a lifestyle, philosophy, it transports values	
Connection to my (local) region	
**With which of the reasons listed below do you justify not to buy organic food?** *(tick all that apply)*	
High Prices	☐
Mistrust in certification	☐
Lack of sufficient availability/accessibility	☐
Prefer local food	☐
Unattractive appearance	☐
Poor assortment of products	☐
Lack of local fresh organic products	☐
I can't distinguish organic food on the market	☐
Lack of sufficient knowledge about benefits of organic food (for health, environment etc.)	☐
None. I always/mainly buy organic food	☐
Other:	☐________________________

**Has the purchase of organic food in your household increased in the last 5 years?**				
☐Yes, very much	☐Yes, slightly	☐No		
If yes – **what was/were the reason/s for this change?** (Tick all that apply)				
☐The prices of organic products have decreased	☐ My awareness about the positive impact of organic production on the environment and animal welfare has increased	☐ My awareness that organic food is better for my health has increased	☐ My awareness that organic food contains less pesticide residues, artificial fertilizers, food additives has increased	☐ I started paying attention to the quality of the food I buy for my family
☐ Due to illness (mine or a family member)	☐ My awareness that organic production is a sustainable production system has increased	☐ Availability of organic food has increased	☐ I have learnt to appreciate the better and natural taste of organic food	☐ Because of pregnancy and the birth of a child
☐ Other: ____				

**food examples need to be adapted to case territory

****need to be adapted to case territory, possibly add examples from local initiatives

**Thank you very much for completing this questionnaire. In case of any comments, you can type these in the space below.**
**If you want to follow the results of the study, please visit our website** SysOrg | SUSFOOD2 ERA-NET (susfood-db-era.net)
