# Supplementary material for: Entry Points, Barriers, and Drivers of Transformation Toward Sustainable Organic Food Systems in Five Case Territories in Europe and North Africa
Source: Nutrients. 2025 Jan 25;17(3):445. doi: 10.3390/nu17030445 (PMC11820227; doi:10.3390/nu17030445)
Supplement: Supplementary file 1 [file nutrients-17-00445-s001.zip › SM_Table S2.pdf]

Table S2. Proposed types of interventions (entry points).

**System characteristics (realms)**	**Parameters**	**Feedback**	**Design**	**Intent**
Proposed types of interventions (entry points) based on own research in five case territories of SysOrg project	**Adjusting parameters to target development of the organic sector by policymakers by:** **Creating the demand for organic** by e.g. integrating organic into public catering and into dietary guidelines. **Promotion of organic through a top-down approach** e.g. local food policy integrating organic, creating local marketplaces for organic producers, support for CSA and cooperatives, subsidies. **Providing training and education** e.g. school education on the benefits of sustainable organic, tailored education for all organic and non-organic stakeholders, training for small-scale farmers on organic farming, training on organic regulations, consultations on starting organic farming.	**Reducing information delay by:** **Strengthening of network between consumers, producers and nature in local ecosystems** by e.g. endorsing local organic farmers with media exposure, media exposure actions in most trending mass communication channels, farm educational visits, farmers markets. **Popularizing negative feedback** by spreading awareness on e.g. unsustainability of conventional farming and its effects on climate change in public institutions. **Strengthening the effectiveness of a given incentives scheme by tailored nudging mechanism** e.g. for consumers driven mainly by health motivations through the information about reducing pesticide residues by organic consumption, for consumers driven by environmental and ethical motivations – the message about the increased level of environmental sustainability or wellbeing of animals in organic production. **Establishing country/regional specific traditional frames that can support developing of organic sector with *positive feedback** - like Beldi foods or local foods **Establishing country/region specific cultural frames** e.g. willingness to change into plant-based.	**Strengthening the feedback by:** **Making the system more inclusive** e.g. by involving organic actors in the public spaces - municipalities, universities, local and central markets (free access to marketplaces, public procurement with organic criteria). **Strengthening of structure of information flows** e.g. access to scientific research about organic food for all stakeholders, access to universities both active and passive for organic stakeholders e.g. lectures conducted by organic farmers, lectures designed for organic stakeholders. **Increase the power to add, change, evolve, or self-organize by:** e.g. participatory approach to certification, a certification process with more involvement of the stakeholders in certification schemes. **Building organic** communities, organic districts, cooperatives with diversity of stakeholders and thoughts**. Increasing biodiversity** in organic farming and diversity in the organic market.	**Targeting the goals of the system by:** **Economic growth** – with the economic growth the trajectory organic transformation is more stable. **Sustainable growth** – while price is a barrier, the most significant drivers are awareness and availability. **Modeling a system and mindset:** **Awareness-building**: promoting "green" mindset - sustainable and ecofriendly lifestyle, developing and promoting a clear research-based definition of a sustainable diet and organic food by tailored educational campaigns raising awareness about benefits of organic both for farmers and consumers, creating forums for farmers and consumers. **The power to transcend paradigms:** **Matching supply and demand by increasing plant-based local organic products** – increasing the quality and nutritional value of food while building consumer trust and recognition
